# Supplementary material for: Genome build information is an essential part of genomic track files
Source: Genome Biol. 2017 Sep 14;18:175. doi: 10.1186/s13059-017-1312-1 (PMC5599886; doi:10.1186/s13059-017-1312-1)
Supplement: Additional file 1: Additional Tables S1 and S2. — (PDF 79 kb) [file 13059_2017_1312_MOESM1_ESM.pdf]

Genome build information in a sample of journals

We employed a preliminary search across a sample of four journals to identify relevant articles with the following criteria: articles published after 2008-12-31; abstract or title should contain any of the species name or its synonyms for *Homo sapiens, Mus musculus, Drosophila melanogaster* AND abstract or title should contain any of the following keywords 'genome', 'epigenome', 'sequencing', 'mapping', 'transcriptome', 'rnaseq', 'chipseq', 'exome sequencing', 'whole genome sequencing'. For all such articles, we checked whether the full text explicitly contained the genome build information, which varied from 16.5% - 52.3% (**Additional Table 1**). Although the search criteria carry a limitation of retrieving some studies that had no real mapping step involved (against a genome build), these statistics at least reveal that genome build information is not always mentioned in the full text of journal articles.

Next, we employed a sequence of filtering steps to shortlist those articles that most likely had a mapping step involved against a reference genome for species of interest. For this, we screened all the retrieved articles for GEO accession IDs (series IDs starting with GSE). If multiple GEO accession IDs are detected in the article, then we examined only one accession ID. We then filtered records matching the following criteria: organisms - *Homo sapiens, Mus musculus, Drosophila melanogaster;* submitted after 2008-12-31; technology platform – high-throughput sequencing. We then examined the consistency of supplying genome build information to both journal and repository (**Additional Table 2**).

Failed cases of prediction with Genome Build Predictor tool

When tested on DNase HSS narrow peak files from Roadmap Epigenomics (n=131), the tool was not able to process ~ 30% of the files that did not strictly adhere to the file format specifications, whereas the prediction failed on ~ 9% of the files as the genomic intervals

contained start/end coordinates that are larger than the size of chromosome-M on hg38, hg19, hg18 and hg17.

Additional Table 1: Genome build information scenario as examined in a sample of four journals

Journal	Total articles screened	Genome build Information In full text
BMC Genomics	1749	499 (28.5%)
PLOS One	5646	917 (16.2%)
Genome Biology	327	170 (51.9%)
Nature Genetics	287	150 (52.2%)

Additional Table 2: Consistency of supplying genome build information to both journal and repository

Journal	Exclusively in GEO database	Exclusively in full text of article	Consistently supplied to both	Supplied to none
BMC GENOMICS	12	23	51	5
GENOME BIOLOGY	7	9	52	3
NATURE GENETICS	5	5	29	1
PLOS ONE	24	18	83	5
Total	48 (14.5%)	55 (16.5%)	215 (64.8%)	14 (4.2%)
